# On Some Approximation Theorems for Power *q*-Bounded Operators on Locally Convex Vector Spaces

**DOI:** 10.1155/2014/513162

**Published:** 2014-08-18

**Authors:** Ludovic Dan Lemle

**Affiliations:** Department of Electrical Engineering and Industrial Informatics, Politehnica University of Timișoara, 331128 Hunedoara, Romania

## Abstract

This paper deals with the study of some operator inequalities involving the power *q*-bounded operators along with the most known properties and results, in the more general framework of locally convex vector spaces.

## 1. Introduction

Let *X* be a Hausdorff locally convex vector space over the complex field *C*. By* calibration* for the locally convex space *X* we understand a family *P* of seminorms generating the topology *τ*_*P*_ of *X*, in the sense that this topology is the coarsest with respect to the fact that all the seminorms in *P* are continuous. Such a family of seminorms was used by the author and Wu [1] and many others in different contexts (see [2–5]).

It is well known that calibration *P* is characterized by the property that the set

(1) $$\begin{array}{c} E\left(p,\epsilon \right)=\left\{x\in X:p\left(x\right)<\epsilon \right\},\;\epsilon >0,\;\;p\in P \end{array}$$

is a neighborhood subbase at 0. Denote by (*X*, *P*) the locally convex space *X* endowed with calibration *P*.

Recall that a locally convex algebra is an algebra with a locally convex topology in which the multiplication is separately continuous. Such an algebra is said to be locally *m*-convex (l.m.c.) if it has a neighborhood base *U* at 0 such that each *U* ∈ *U* is convex and balanced (i.e., *λU*⊆*U* for |*λ* | ≤1) and satisfies the property *U* ^2^⊆*U*.

Any algebra with identity will be called unital. It is well known that unital locally *m*-convex algebra *A* is characterized by the existence of calibration *P* such that each *p* ∈ *P* is submultiplicative (i.e., *p*(*xy*) ≤ *p*(*x*)  *p*(*y*), for all *x*, *y* ∈ *A*) and satisfies *p*(*e*) = 1, where *e* is the unit element.

An element *a* of locally convex algebra *A* is said to be* bounded in A* if there exists *α* ∈ *C* such that the set {(*αx*)^*n*^}_*n*≥1_ is bounded in *A* (see [6]). The set of all bounded elements in *A* will be denoted by *A*_0_.

Let *C*_*∞*_ : = *C* ∪ {*∞*} be the Alexandroff one-point compactification of *C*. Following Waelbroeck [7, 8], we introduce the following.

**Definition 1 .**

We call resolvent set in the Waelbroeck sense of an element *x* from a locally convex unital algebra (*X*, *P*) the set of all elements *λ*_0_ ∈ *C*_*∞*_ for which there exists *V* ∈ *V*_*λ*_0__ such that the following conditions hold:

- the element *λe* − *x* is invertible in *X*, for any *λ* ∈ *V*∖{*∞*};
- the set {(*λe* − *x*)^−1^ : *λ* ∈ *V*∖{*∞*}} is bounded in (*X*, *P*).

The resolvent set in Waelbroeck sense of an element *x* will be denoted by *ρ*_*W*_(*x*). The* Waelbroeck spectrum* of *x* will be defined as

(2) $$\begin{array}{c} \sigma_{W}\left(x\right):=C_{\infty }\setminus \rho_{W}\left(x\right). \end{array}$$

## 2. *q*-Bounded Operators

Following Michael [9] (see also [2, 10]), we introduce the following.

**Definition 2 .**

We say that a linear operator *T* : *X* → *X* is *q*-bounded (quotient-bounded) with respect to *P* if for any *p* ∈ *P* there exists *c*_*p*_ > 0 such that

(3) $$\begin{array}{c} p\left(Tx\right)\leq c_{p}p\left(x\right),\;\forall x\in X. \end{array}$$

Denote by *Q*_*P*_(*X*) the set which consists of all *q*-bounded operators with respect to calibration *P*.

For a seminorm *p* ∈ *P*, the application $\hat{p}:Q_{P}(X)\to R$ defined as

(4) $$\begin{array}{c} \hat{p}\left(T\right)=\inf\left\{r>0:p\left(Tx\right)\leq rp\left(x\right),\;\;\forall x\in X\right\} \end{array}$$

is also a seminorm. Note that

(5) $$\begin{array}{c} \hat{p}\left(T_{1}T_{2}\right)\leq \hat{p}\left(T_{1}\right)\hat{p}\left(T_{2}\right),\;T_{1},T_{2}\in Q_{P}\left(X\right),\;\;p\in P. \end{array}$$

We denote by $\hat{P}$ the family of seminorms $\left\{\hat{p}:p\in P\right\}$. The space *Q*_*P*_(*X*) will be endowed with a topology $\tau_{\hat{P}}$ generated by $\hat{P}$. Remark that [9, Proposition 2.4(j)] implies that under this topology *Q*_*P*_(*X*) becomes a Hausdorff locally *m*-convex topological algebra (in the sense of [9, Definition 2.1]).

If *T* ∈ *Q*_*P*_(*X*), the *P*-spectral radius, denoted by *r*_*P*_(*T*), is considered as the boundedness radius in the sense of Allan [6] (see also [11–13]),

(6) rP(T)=inf⁡{λ>0:the  sequence ((λ−1T)n)n∈Nis  bounded  in  QP(X)},

where, by common consent, inf⁡*∅* : = +*∞*.

The set of all bounded elements in *Q*_*P*_(*X*) will be denoted by (*Q*_*P*_(*X*))_0_ (see [12]). It easily follows from [6, Proposition 2.14(ii)] that

(7) $$\begin{array}{c} {\left(Q_{P}\left(X\right)\right)}_{0}=\left\{T\in Q_{P}\left(X\right):r_{P}\left(T\right)<\infty \right\}. \end{array}$$

For *T* ∈ (*Q*_*P*_(*X*))_0_ we denote by *ρ*_*W*_(*T*) the* Waelbroeck resolvent set of T* and by *σ*_*W*_(*T*) the* Waelbroeck spectrum of T*. The function

(8) $$\begin{array}{c} \rho_{W}\left(T\right)∋\lambda ⟼R\left(\lambda ,T\right)∶={\left(\lambda I-T\right)}^{-1}\in {\left(Q_{P}\left(X\right)\right)}_{0} \end{array}$$

is called the* resolvent function of T*. It is well known that

(9) $$\begin{array}{c} R\left(\lambda ,T\right)=\overset{\infty }{\underset{n=0}{\sum }}\frac{T^{n}}{\lambda^{n+1}}. \end{array}$$

In this paper we evaluate the behaviour of the power of a *q*-bounded operator from the algebra (*Q*_*P*_(*X*))_0_ by some type of approximations. The main results have been announced in [14].

## 3. The Main Results

We continue to employ the notations from the previous sections and we will introduce two types of operatorial approximations for operators from the algebra (*Q*_*P*_(*X*))_0_ which approximate a given operator *T* on a convergent power bounded series. The power boundedness problem for operators acting on Banach spaces was largely developed in various frameworks by many authors (see [15–17]).

In the following, using the functional calculus from the (*Q*_*P*_(*X*))_0_ algebra (see [7, 8]), some important boundedness properties are obtained. Denote N* = *N*∖{0}. First we have the following.

**Theorem 3 .**

If *T* ∈ (*Q*_*P*_(*X*))_0_ satisfies

(10) $$\begin{array}{c} \underset{\hat{p}\in \hat{P}}{\sup}\hat{p}\left(T^{k}\right)\leq C, \end{array}$$

for *k* ∈ N*, then

(11) $$\begin{array}{c} \underset{\hat{p}\in \hat{P}}{\sup}\hat{p}\left[R{\left(\lambda ,T\right)}^{k}\right]\leq \frac{C}{{\left(\left|\lambda \right|-1\right)}^{k}}, \end{array}$$

for *k* ∈ N* and for all *λ* ∈ *C* with |*λ* | >1.

**Proof**

Assume that $\sup_{\hat{p}\in \hat{P}}\hat{p}(T^{k})\leq C$ for *k* ∈ N*. Since

(12) $$\begin{array}{c} R\left(\lambda ,T\right)=\overset{\infty }{\underset{j=0}{\sum }}\frac{T^{j}}{\lambda^{j+1}}, \end{array}$$

for |*λ* | >1, then, by using the generalized binomial formula, we get

(13) $$\begin{array}{c} R{\left(\lambda ,T\right)}^{k}=\lambda^{-k}{\left(I-\frac{T}{\lambda }\right)}^{-k}=\frac{1}{\lambda^{k}}\overset{\infty }{\underset{j=0}{\sum }}\left(\begin{array}{c} j+k-1\\ j \end{array}\right)\frac{T^{j}}{\lambda^{j}}, \end{array}$$

from where we deduce

(14) p^[R(λ,T)k]≤C|λ|k·∑j=0∞(j+k−1j)(1|λ|)j=C|λ|k·1(1−1/|λ|)k=C(|λ|−1)k,

for any *k* ∈ N* and any $\hat{p}\in \hat{P}$. Therefore, the conclusion is verified.

Conversely, we have the following.

**Theorem 4 .**

If *T* ∈ (*Q*_*P*_(*X*))_0_ and

(15) $$\begin{array}{c} \underset{\hat{p}\in \hat{P}}{\sup}\hat{p}\left[R\left(\lambda ,T\right)\right]\leq \frac{C}{\left|\lambda \right|-1}, \end{array}$$

for all *λ* ∈ *C* with |*λ* | >1, then

(16) $$\begin{array}{c} \underset{\hat{p}\in \hat{P}}{\sup}\hat{p}\left(T^{k}\right)\leq Ce\left(k+1\right), \end{array}$$

for *k* ∈ N*.

**Proof**

Let us suppose condition $\hat{p}[R(\lambda ,T)]\leq C/\left(|\lambda |-1\right)$ is true for all $\hat{p}\in \hat{P}$, for any *k* ∈ N* and |*λ* | >1. For *k* ∈ N* fixed, by choosing the integration path Γ : |*λ* | = 1 + 1/*k*, with the aid of the functional calculus from the algebra (*Q*_*P*_(*X*))_0_, we obtain

$$\begin{array}{cc} \left(*\right) & T^{k}=\frac{1}{2\pi i}\overset{}{\underset{\Gamma }{\int }}\lambda^{k}R\left(\lambda ,T\right)d\lambda . \end{array}$$

Thus, for all $\hat{p}\in \hat{P}$, we have

(17) p^(Tk)≤12π∫Γ|λ|kp^(R(λ,T))dλ≤12π·max⁡λ∈Γ|λ|k·max⁡λ∈ΓC|λ|−1·∫Γdλ≤12π·(1+1k)k·Ck·2π(1+1k)≤Ce(k+1)

which implies the desired result.

Moreover, we can formulate the following.

**Theorem 5 .**

If *T* ∈ (*Q*_*P*_(*X*))_0_ and

(18) $$\begin{array}{c} \underset{\hat{p}\in \hat{P}}{\sup}\hat{p}\left[R{\left(\lambda ,T\right)}^{k}\right]\leq \frac{C}{{\left(\left|\lambda \right|-1\right)}^{k}}, \end{array}$$

for *k* ∈ N* and for all *λ* ∈ *C* with |*λ* | >1, then

(19) $$\begin{array}{c} \underset{\hat{p}\in \hat{P}}{\sup}\hat{p}\left(T^{k}\right)\leq C\frac{k!e^{k}}{k^{k}}\leq C\sqrt{2\pi \left(k+1\right)},\;k\in N^{*}. \end{array}$$

**Proof**

Integrating *(∗)* by parts *j* − 1 times, for *j* > 2, we obtain

(20) $$\begin{array}{c} T^{k}=\frac{{\left(-1\right)}^{j-1}}{2\pi i}\overset{}{\underset{\Gamma }{\int }}\frac{\left(j+1\right)!\lambda^{k+j-1}}{\left(k+1\right)\cdots \left(k+j-1\right)}R{\left(\lambda ,T\right)}^{j}d\lambda . \end{array}$$

Now choosing Γ the circle of radius 1 + *j*/*k* and by using the hypothesis, for *j* → *∞*, we get

(21) $$\begin{array}{c} \underset{\hat{p}\in \hat{P}}{\sup}\hat{p}\left(T^{k}\right)\leq C\frac{k!e^{k}}{k^{k}}\leq C\sqrt{2\pi \left(k+1\right)}. \end{array}$$

The last inequality was obtained by using Stirling's approximation.

Now, for *T* ∈ (*Q*_*P*_(*X*))_0_ we introduce (see [18]) the following.

**Definition 6 .**

The Yosida approximation *Y*(*λ*, *T*) of *T*, for *λ* ∈ *ρ*_*W*_(*T*)∩*C*, is defined as

(22) $$\begin{array}{c} Y\left(\lambda ,T\right)=\lambda TR\left(\lambda ,T\right). \end{array}$$

Next theorem shows how an operator from the (*Q*_*P*_(*X*))_0_ algebra is related to its Yosida approximation.

**Theorem 7 .**

The Yosida approximation *Y*(*λ*, *T*) is analytic for *λ* ∈ *ρ*_*W*_(*T*)∩*C* and the series representation

(23) $$\begin{array}{c} Y\left(\lambda ,T\right)=\overset{\infty }{\underset{j=0}{\sum }}\frac{T^{j+1}}{\lambda^{j}} \end{array}$$

converges for |*λ* | >*r*_*P*_(*T*). Moreover,

(1) *Y*(*λ*, *T*) = *λ* ^2^ *R*(*λ*, *T*) − *λI*;
(2) if there exists $\hat{p}\in \hat{P}$ such that $r_{P}(T)<\hat{p}(T)$, then
  (24) $$\begin{array}{c} \hat{p}\left(Y\left(\lambda ,T\right)-T\right)\leq \frac{\hat{p}\left(T^{2}\right)}{|\lambda |-\hat{p}\left(T\right)}, \end{array}$$
  for $\left|\lambda |>\hat{p}(T\right)$;
(3) *σ*_*W*_(*Y*(*λ*, *T*)) = {*z*/(1 − *z*/*λ*), *z* ∈ *σ*_*W*_(*T*)}.

**Proof**

By evaluating *Y*(*λ*, *T*) in terms of the resolvent *R*(*λ*, *T*), for |*λ* | >*r*_*P*_(*T*) we obtain

(25) Y(λ,T)=λTR(λ,T)=λT(λI−T)−1=λT·∑j=0∞Tjλj+1=∑j=0∞Tj+1λj

from where it follows that the assertion of the theorem is true. Moreover,

(26) Y(λ,T)=∑j=0∞Tj+1λj=λI+T+T2λ+⋯+Tn+1λn+⋯−λI=λ2∑j=0∞Tjλj+1−λI=λ2R(λ,T)−λI,

so (1) is true.

To prove (2) one can observe that, from

(27) $$\begin{array}{c} Y\left(\lambda ,T\right)=\overset{\infty }{\underset{j=0}{\sum }}\frac{T^{j+1}}{\lambda^{j}}, \end{array}$$

it follows that

(28) $$\begin{array}{c} Y\left(\lambda ,T\right)-T=\overset{\infty }{\underset{j=0}{\sum }}\frac{T^{2}}{\lambda }\left(\frac{T^{j}}{\lambda^{j}}\right) \end{array}$$

on a set for which |*λ* | >*r*_*P*_(*T*). Moreover,

(29) p^(Y(λ,T)−T)≤∑j=0∞p^(T2λ)p^(Tjλj)≤p^(T2λ)·∑j=0∞p^(Tλ)j=p^(T2λ)11−p^(T/λ)=p^(T2)|λ|−p^(T),

for $\left|\lambda |>\hat{p}(T)>r_{P}(T\right)$.

A simple reasoning shows that *R*(*λ*, *T*)∈(*Q*_*P*_(*X*))_0_; then it follows *Y*(*λ*, *T*)∈(*Q*_*P*_(*X*))_0_.

From [19, Theorem 3.1.14], for |*λ* | >|*z*|, we have

(30) $$\begin{array}{c} \sigma_{W}\left(Y\left(\lambda ,T\right)\right)=Y\left(\lambda ,\sigma_{W}\left(T\right)\right), \end{array}$$

for all *z* ∈ *σ*_*W*_(*T*), and

(31) $$\begin{array}{c} Y\left(\lambda ,z\right)=\overset{\infty }{\underset{j=0}{\sum }}\frac{z^{j+1}}{\lambda^{j}} \end{array}$$

on |*λ* | >|*z*|, which could be written as *Y*(*λ*, *z*) = *z*/(1 − *z*/*λ*), for any *z* ∈ *σ*_*W*_(*T*), so (3) is proved.

Below we state an equivalence between a power bounded operator from the (*Q*_*P*_(*X*))_0_ algebra and the power of its Yosida approximation.

**Theorem 8 .**

Let *T* ∈ (*Q*_*P*_(*X*))_0_ and *Y*(*λ*, *T*) its Yosida approximation. Then the following assertions are equivalent:

- $\sup_{\hat{p}\in \hat{P}}\hat{p}(T^{k})\leq c$, for any *k* ∈ N*;
- $\sup_{\hat{p}\in \hat{P}}\hat{p}(Y{\left(\lambda ,T\right)}^{k})\leq c/{\left(1-1/\left|\lambda \right|\right)}^{k}$, for any *k* ∈ N* and for all *λ* ∈ *C* with |*λ* | >1.

**Proof**

Property (i) implies *r*_*P*_(*T*) ≤ 1 so that the argumentation given in the proof of Theorem 7 implies that any *λ* ∈ *C* with |*λ* | >1 belongs to the resolvent set of *T*. Hence, using the generalized binomial formula, we get

(32) $$\begin{array}{c} Y{\left(\lambda ,T\right)}^{k}=\overset{\infty }{\underset{j=0}{\sum }}\left(\begin{array}{c} k+j-1\\ j \end{array}\right)\frac{T^{j+k}}{\lambda^{j}}. \end{array}$$

Now, by applying (i) again we obtain

(33) $$\begin{array}{c} \hat{p}\left(Y{\left(\lambda ,T\right)}^{k}\right)\leq c\overset{\infty }{\underset{j=0}{\sum }}\left(\begin{array}{c} k+j-1\\ j \end{array}\right){\left(\frac{1}{\left|\lambda \right|}\right)}^{j}=\frac{c}{{\left(1-1/\left|\lambda \right|\right)}^{k}} \end{array}$$

for any $\hat{p}\in \hat{P}$, whence by passing to supremum, the inequality (ii) holds.

Conversely, (i) is a direct consequence of (ii).

For *μ* ∈ *ρ*_*W*_(*T*), consider now the following* Möbius transformation* (see [20]):

(34) $$\begin{array}{c} \psi_{\lambda }\left(\mu \right)=\{\begin{array}{cc} \frac{\left(\lambda -1\right)\mu }{\lambda -\mu }, & \text{if}\;\;\lambda \neq \infty ,\\ \mu , & \text{if}\;\;\lambda =\infty . \end{array} \end{array}$$

**Definition 9 .**

The Möbius approximation of *T* is defined as

(35) $$\begin{array}{c} A\left(\lambda ,T\right):=\psi_{\lambda }\left(T\right). \end{array}$$

**Proposition 10 .**

*A*(*λ*, *T*) is holomorphic in *λ* ∈ *ρ*_*W*_(*T*) ∩ *C* and satisfies

(36) $$\begin{array}{c} A\left(\lambda ,T\right)=\left(1-\frac{1}{\lambda }\right)Y\left(\lambda ,T\right),\;\;\;\lambda \neq 0. \end{array}$$

**Proof**

Let *λ* ∈ *ρ*_*W*_(*T*) ∩ *C*∖{0}. By evaluating the right member of the above equality, we get successively

(37) (1−1λ)Y(λ,T) =(1−1λ)λTR(λ,T)=(λ−1)TR(λ,T) =(λ−1)TλI−T=A(λ,T)

for *λ* ≠ *∞*. If *λ* = *∞*, then from Definition 9 we have *A*(*λ*, *T*) = *T*. On the other side (1 − 1/*λ*)*Y*(*λ*, *T*) converges to *T*, when *λ* → *∞*.

A similar result as in Theorem 8 is given below.

**Theorem 11 .**

Let *T* ∈ (*Q*_*P*_(*X*))_0_ and *A*(*λ*, *T*) its approximation as above. Then the following assertions are equivalent:

- $\sup_{\hat{p}\in \hat{P}}\hat{p}(T^{k})\leq C$, for any *k* ∈ N*;
- $\sup_{\hat{p}\in \hat{P}}\hat{p}(A{\left(\lambda ,T\right)}^{k})\leq C$, for any *k* ∈ N* and for every *λ* ∈ *C* with |*λ* | >1.

**Proof**

From Theorem 8, for *T* ∈ (*Q*_*P*_(*X*))_0_,

(38) $$\begin{array}{c} \underset{\hat{p}\in \hat{P}}{\sup}\hat{p}\left(T^{k}\right)\leq C \end{array}$$

is equivalent to

(39) $$\begin{array}{c} \underset{\hat{p}\in \hat{P}}{\sup}\hat{p}\left(Y{\left(\lambda ,T\right)}^{k}\right)\leq \frac{C}{{\left(1-1/\left|\lambda \right|\right)}^{k}}. \end{array}$$

The conclusion follows taking into account that

(40) $$\begin{array}{c} A{\left(\lambda ,T\right)}^{k}={\left(1-\frac{1}{\lambda }\right)}^{k}\cdot Y{\left(\lambda ,T\right)}^{k}, \end{array}$$

for *k* ∈ N*.

## 4. Application

For *L* > 0 let *X* : = *C*[0, *L*] be the space of continuous functions on [0, *L*] endowed with the norm |*u*|_*L*_ : = max⁡_[0,*L*]_ | *u*(*t*)|.

Consider *T* : *X* → *X*, given by

(41) $$\begin{array}{c} Tu\left(t\right)=\overset{t}{\underset{0}{\int }}u\left(s\right)ds. \end{array}$$

Following [19], we see that the resolvent of *T* is given by

(42) $$\begin{array}{c} R\left(\lambda ,T\right)u\left(t\right)=\frac{1}{\lambda }u\left(t\right)+\frac{1}{\lambda^{2}}\overset{t}{\underset{0}{\int }}e^{(t-s)/\lambda }u\left(s\right)ds, \end{array}$$

the Yosida approximation of *T* is

(43) $$\begin{array}{c} Y\left(\lambda ,T\right)u\left(t\right)=\overset{t}{\underset{0}{\int }}e^{(t-s)/\lambda }u\left(s\right)ds, \end{array}$$

and the Möbius approximation of *T* is

(44) $$\begin{array}{c} A\left(\lambda ,T\right)u\left(t\right)=\left(1-\frac{1}{\lambda }\right)\overset{t}{\underset{0}{\int }}e^{(t-s)/\lambda }u\left(s\right)ds. \end{array}$$

Remark that, for all *u* ∈ *C*[0, *L*], we have

(45) |Tu|L=max⁡t∈[0,L]|Tu(t)|≤max⁡t∈[0,L]∫0t|u(s)|ds≤max⁡t∈[0,L]|u(t)|∫0Lds=|u|L·L.

The above implies that *T* is a contraction for *L* ≤ 1.

If *L* > 1, then we can introduce for each *ɛ* > 0 the following norm on *C*[0, *L*]:

(46) $$\begin{array}{c} {||u||}_{ɛ}:=\underset{t\in [0,L]}{\max}e^{t/ɛ}\left|u\left(t\right)\right|,\;\;\;u\in C\left[0,L\right]. \end{array}$$

Then a simple computation gives that

(47) $$\begin{array}{c} {||Tu||}_{ɛ}<ɛ{||u||}_{ɛ},\;\;\;u\in C\left[0,L\right]. \end{array}$$

On the other hand,

(48) $$\begin{array}{c} {||u||}_{ɛ}\leq {\left|u\right|}_{L}\leq e^{L/ɛ}{||u||}_{ɛ}. \end{array}$$

Remark that, by Theorem 11, for all *λ* > 1, we get

(49) $$\begin{array}{c} {\left|A\left(\lambda ,T\right)\right|}_{L}=\left(\lambda -1\right)\left(e^{T/\lambda }-1\right)\leq 1 \end{array}$$

if and only if |*T*|_*L*_ ≤ 1.

It is clear that for estimating the powers of *T* it seems to be better to use the Yosida approximation or Möbius approximation than the resolvent approximation.

## References

[1] Lemle LD, Wu LM (2011). Uniqueness of C_0_-semigroups on a general locally convex vector space and an application. *Semigroup Forum*.

[2] Moore RT (1969). Banach algebras of operators on locally convex spaces. *Bulletin of the American Mathematical Society*.

[3] Pater F (2011). Properties of multipliers on special algebras with application to signal processing. *Acta Technica Napocensis: Applied Mathematics and Mechanics*.

[4] Pater F A multiplier algebra representation with application to harmonic signal models.

[5] Kostić M (2012). Abstract Volterra equations in locally convex spaces. *Science China: Mathematics*.

[6] Allan GR (1965). A spectral theory for locally convex alebras. *Proceedings of the London Mathematical Society*.

[7] Waelbroeck L (1960). *Étude Spectrale des Algèbres Complètes*.

[8] Waelbroeck L (1957). Algébrés commutatives: éléments réguliers. *Bulletin of the Belgian Mathematical Society*.

[9] Michael EA (1952). Locally multiplicatively-convex topological algebras. *Memoirs of the American Mathematical Society*.

[10] Joseph GA (1977). Boundedness and completeness in locally convex spaces and algebras. *Journal of the Australian Mathematical Society*.

[11] Bonales FG, Mendoza RV (1998). Extending the formula to calculate the spectral radius of an operator. *Proceedings of the American Mathematical Society*.

[12] Pater F, Binzar T (2010). On some ergodic theorems for a universally bounded operator. *Carpathian Journal of Mathematics*.

[13] Pater F, Lemle LD On some multiplication operator algebra problem with application to stochastic signal models.

[14] Pater F, Lemle LD, Binzar T On some Yosida type approximation theorems.

[15] Katznelson Y, Tzafriri L (1986). On power bounded operators. *Journal of Functional Analysis*.

[16] Nagy B, Zemanek J (1999). A resolvent condition implying power boundedness. *Studia Mathematica*.

[17] Nevanlinna O (1997). On the growth of the resolvent operator for power bounded operators, Linear Operators. *Banach Center Publications*.

[18] Yosida K (1948). On the differentiability and the representation of one-parameter semi-group of linear operators. *Journal of the Mathematical Society of Japan*.

[19] Nevanlinna O (1993). *Convergence of Iterations for Linear Equations*.

[20] Shields AL (1978). On Möbius bounded operators. *Acta Universitatis Szegediensis: Acta Scientiarum Mathematicarum*.

